# Supplementary material for: TNK1 is a ubiquitin-binding and 14-3-3-regulated kinase that can be targeted to block tumor growth
Source: Nat Commun. 2021 Sep 9;12:5337. doi: 10.1038/s41467-021-25622-3 (PMC8429728; doi:10.1038/s41467-021-25622-3)
Supplement: Supplementary file 1 — Supplementary Information [file 41467_2021_25622_MOESM1_ESM.pdf]

a

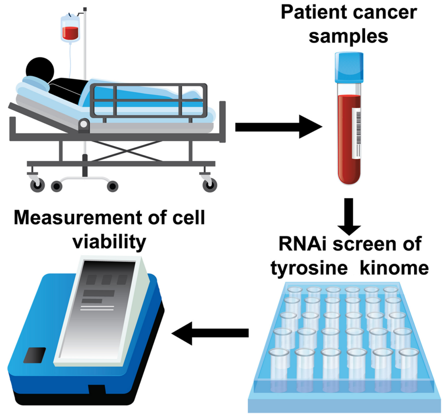

b

Frequency of TNK1 as the top hit among all tyrosine kinases tested

Cancer type	Fraction	Percentage
AML	8/320	2.5%
ALL	6/115	5.2%

c

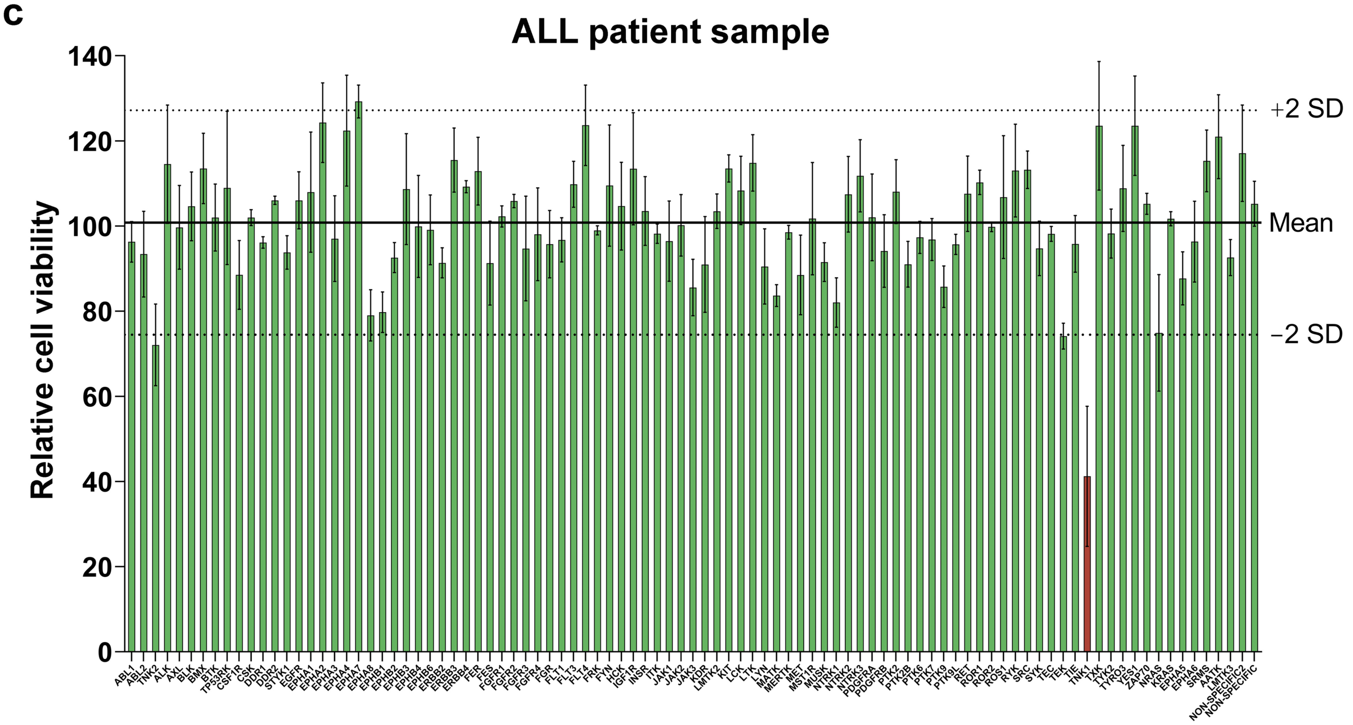

d

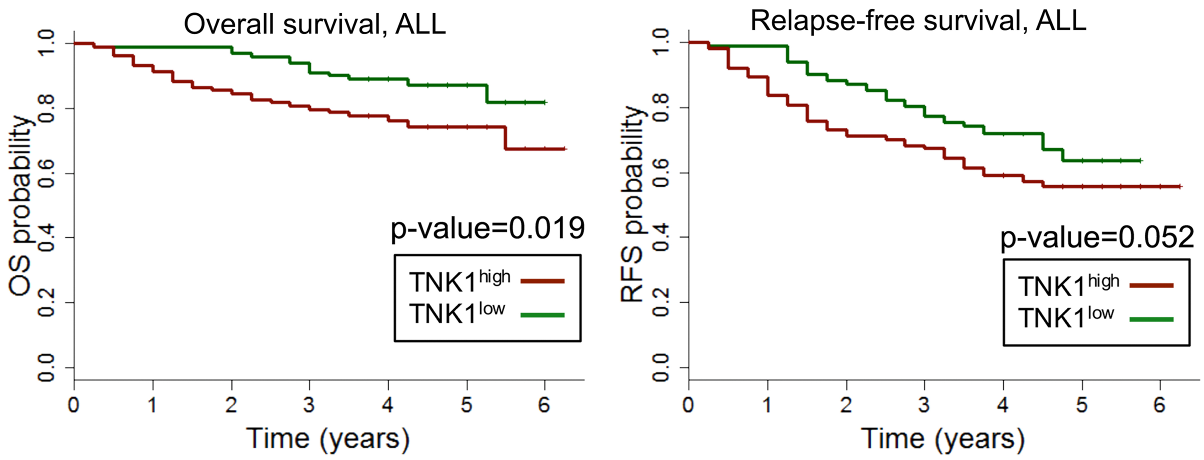

Supplementary Figure 1. RNAi screening of tyrosine kinases across human hematological cancer patient samples reveals a dependency on the non-receptor tyrosine kinase TNK1.

a. Schematic diagram of RNAi screening of tyrosine kinases across 435 hematological cancer samples from human patients. Samples were subjected to an RNAi screen of human tyrosine kinases, followed by measurement of cell viability.
b. Frequency of TNK1 as the top hit (defined as the tyrosine kinase that, when depleted, has the largest negative impact on cell survival in a given sample) from the RNAi screening of tyrosine kinases in AML and ALL. Hit threshold is defined as 2 standard deviations below the mean viability. Error bars represent SEM.
c. A representative viability assay from an ALL patient sample showing TNK1 as the top hit.
d. Kaplan Meier graphs showing correlations between TNK1 mRNA levels and overall survival and relapse-free survival in ALL patients (COG P9906 B-ALL trial, n=207). The data are derived from gene expression microarray data of 207 pre-B ALL patients in the Children's Oncology Group (COG) Clinical Trial P9906. Patient survival data were obtained from the National Cancer Institute TARGET Data Matrix (https://ocg.cancer.gov/programs/target/data-matrix).

a

Uniprot ID	Gene Name	Protein Name
P31946	YWHAB	14-3-3 protein beta/alpha
P62258	YWHAE	14-3-3 protein epsilon
P61981	YWHAG	14-3-3 protein gamma
Q04917	YWHAH	14-3-3 protein eta
Q04917	YWHAQ	14-3-3 protein theta
P63104	YWHAZ	14-3-3 protein zeta/delta

b

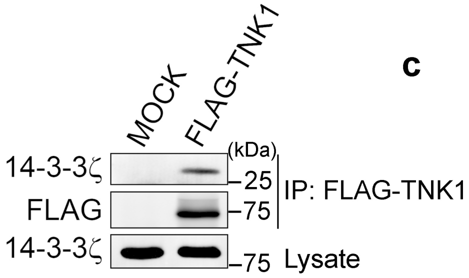

c

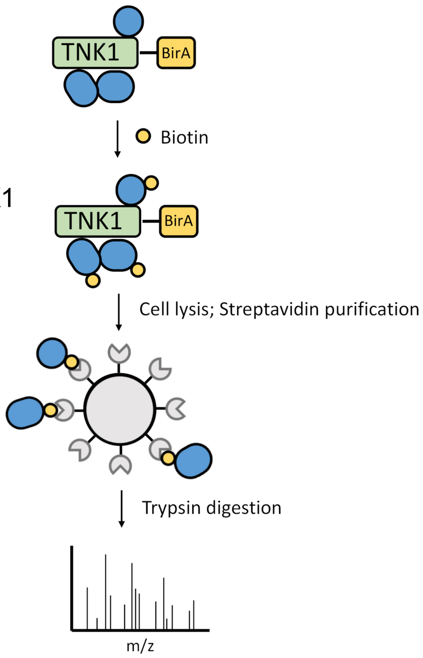

d

Uniprot ID	Gene Name	Protein Name
**Autophagy**		
Q2TAZ0	ATG2A	Autophagy-related protein 2 homolog A
Q96BY7	ATG2B	Autophagy-related protein 2 homolog B
Q13501	SQSTM1	Sequestosome-1 (p62)
Q9UHD2	TBK1	Serine/threonine-protein kinase TBK1
**Migration**		
P55196	AFDN	Afadin
Q14247	CTTN	Src substrate cortactin
Q15417	CNN3	Calponin-3
Q14126	DSG2	Desmoglein-2
Q15691	MAPRE1	Microtubule-associated protein RP/EB family member 1
Q8WX93	PALLD	Palladin
P49023	PXN	Paxillin
**Pro-growth**		
O43318	MAP3K7	Mitogen-activated protein kinase kinase kinase 7
O95819	MAP4K4	Mitogen-activated protein kinase kinase kinase kinase 4
P16333	NCK1	Cytoplasmic protein NCK1
O43639	NCK2	Cytoplasmic protein NCK2
Q04206	RELA	Transcription factor p65/ NF-kappa-B
Q13043	STK4	Serine/threonine-protein kinase 4
P40763	STAT3	Signal transducer and activator of transcription 3
Q15750	TAB1	TGF-beta-activated kinase 1 and MAP3K7-binding protein 1
**Protein Trafficking**		
P42566	EPS15	Epidermal growth factor receptor substrate 15
Q15436	SEC23A	Protein transport protein Sec23A
Q15437	SEC23B	Protein transport protein Sec23B
O95486	SEC24A	Protein transport protein Sec24A
O95487	SEC24B	Protein transport protein Sec24B
Q9Y5X1	SNX9	Sorting nexin-9
**14-3-3 protein**		
P31946	YWHAB	14-3-3 protein beta/alpha
P62258	YWHAE	14-3-3 protein epsilon
P61981	YWHAG	14-3-3 protein gamma
Q04917	YWHAH	14-3-3 protein eta
Q04917	YWHAQ	14-3-3 protein theta
P63104	YWHAZ	14-3-3 protein zeta/delta

e

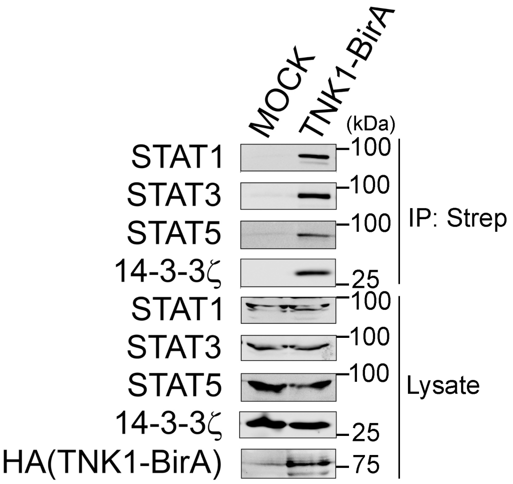

f

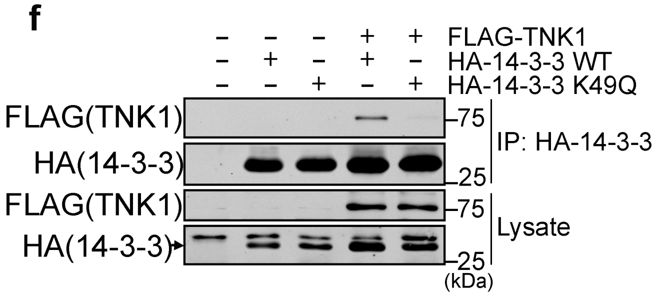

Supplementary Figure 2. Co-IP and BioID proteomics identify 14-3-3 as an interacting partner of TNK1.

a. FLAG-TNK1 was expressed in HEK-293T cells, immunoprecipitated on FLAG resin, followed by elution of interacting partners and analysis by LC-MS/MS. Table shows all 14-3-3 isoforms, except sigma, as TNK1 interactors.
b. FLAG-TNK1 was immunoprecipitated as in panel a, then subject to immunoblot with indicated antibodies. Blot shows a representative image from 3 biological replicates.
c. Schematic of TNK1 BioID.
d. TNK1-BirA was expressed in HEK-293T cells supplemented with biotin, followed by isolation of biotinylated proteins on streptavidin resin and analysis by LC-MS/MS. Table shows TNK1 interacts with autophagy, migration, proliferation and protein trafficking- related proteins.
e. Streptavidin pulldowns from panel d were immunoblotted with indicated antibodies. Blot shows a representative image from 3 biological replicates.
f. HA-14-3-3 (WT or K49Q) was co-expressed with FLAG-TNK1 in HEK-293T cells, followed by immunoprecipitation of HA-14-3-3 and immunoblotting with indicated antibodies. n=3 for 14-3-3 WT; n=1 for 14-3-3 K49Q.

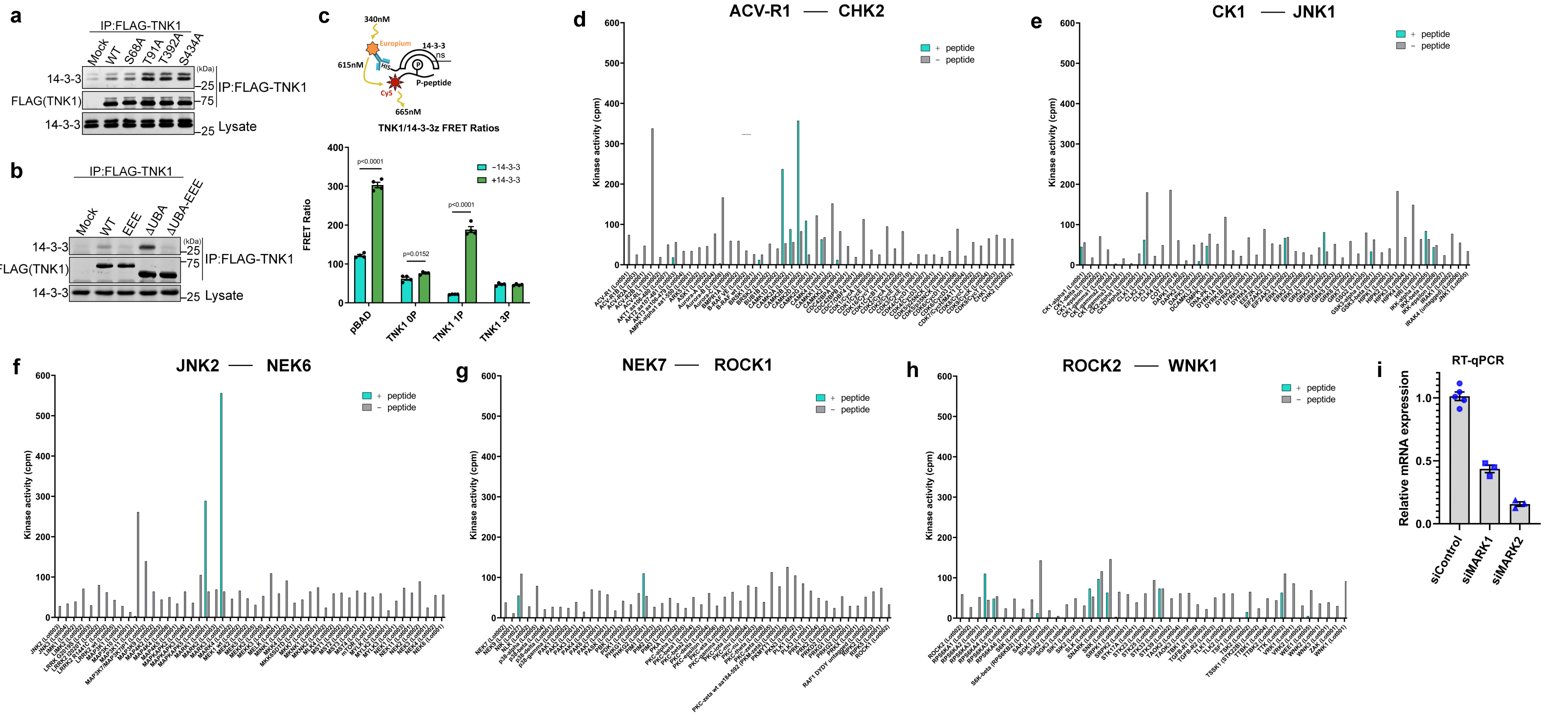

Supplementary Figure 3. Characterization of the 14-3-3 docking phosphorylation on TNK1 and identification of the TNK1 S502-directed kinase.

a. WT or S/T-to-A mutants of FLAG-TNK1 were expressed in HEK-293T cells, immunoprecipitated on FLAG resin and immunoblotted with indicated antibodies. Blot shows representative image from 3 biological replicates.

b. WT or S-to-E triple mutant (S500E/S502E/S505E) of FLAG-TNK1 were expressed in HEK-293T cells, immunoprecipitated on FLAG resin and immunoblotted with indicated antibodies (note: some antibodies to 14-3-3 show a doublet, others a single band). Blots shows representative image from 3 biological replicates.

c. Schematic representation of FRET assay monitoring interaction between 14-3-3 and TNK1 peptide (amino acid 488-511). TNK1 0P peptide has no phosphorylation. TNK1 1P peptide is mono-phosphorylated at S502. TNK1 3P peptide contains phosphorylations on S500, S502 and S505. Graph shows average FRET ratios with error bars representing SEM (n=4 independent experiments). P-values were calculated using two-tailed student t-test.

d.- h. An N-terminally biotinylated 13-mer TNK1 peptide encompassing S502 was incubated in a streptavidin-coated FlashPlateTM and subjected to a radiometric kinase assay with 245 individual S/T protein kinases. To account for signal from kinase autophosphorylation, each assay (+ peptide) was normalized to a control with the kinase but no peptide.

i. RT-qPCR was performed to validate MARK1 and MARK2 knockdown. Graph shows average relative mRNA expression (2^-ΔΔCt) with error bars representing SEM from 3 technical replicates.

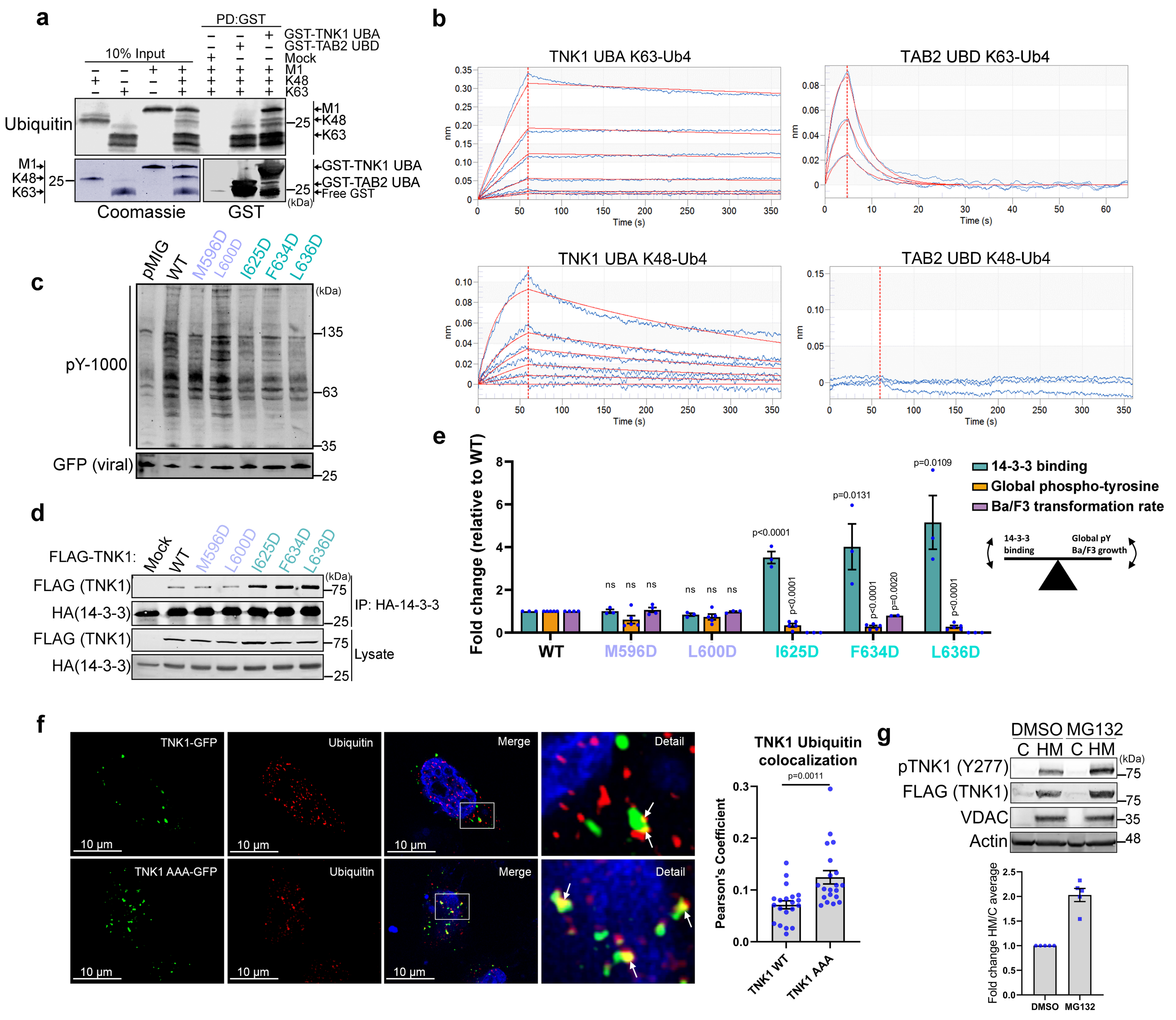
a
PD:GST
10% Input
GST-TNK1 UBA
GST-TAB2 UBD
Mock
M1
K48
K63
Ubiquitin
Coomassie
GST
GST-TNK1 UBA
GST-TAB2 UBA
Free GST
(kDa)
b
TNK1 UBA K63-Ub4
TAB2 UBD K63-Ub4
TNK1 UBA K48-Ub4
TAB2 UBD K48-Ub4
Time (s)
nm
c
pMIG
WT
M596D
L600D
I625D
F634D
L636D
pY-1000
GFP (viral)
(kDa)
d
FLAG-TNK1:
Mock
FLAG (TNK1)
HA(14-3-3)
IP: HA-14-3-3
Lysate
e
Fold change (relative to WT)
14-3-3 binding
Global phospho-tyrosine
Ba/F3 transformation rate
ns
p<0.0001
p=0.0131
p=0.0109
p=0.0020
Global pY
Ba/F3 growth
f
TNK1-GFP
Ubiquitin
Merge
Detail
TNK1 AAA-GFP
10 µm
TNK1 Ubiquitin colocalization
p=0.0011
Pearson's Coefficient
TNK1 WT
TNK1 AAA
g
DMSO
MG132
C
HM
pTNK1 (Y277)
FLAG (TNK1)
VDAC
Actin
Fold change HM/C average
DMSO
MG132

Supplementary Figure 4. TNK1 UBA domain binds poly-ubiquitin with a high affinity, is essential for full TNK1 activity, and toggles TNK1 between 14-3-3-bound and active states.

a. Recombinant GST-TNK1-UBA or GST-TAB2-UBD were incubated with the combined indicated tetra-ubiquitin for a competitive pull-down experiment at 4oC for 2 hours. GST tagged proteins were captured on glutathione resin and immunoblotted for GST and ubiquitin. A 10% input of tetra-ubiquitin for each linkage type was also visualized by Coomassie staining of SDS-PAGE gel to indicate differences in gel migration of the linkages. Blot shows a representative image from 3 biological replicates.
b. Bio-layer interferometry (BLI) was performed to characterize the binding between TNK1-UBA/ TAB2-UBD and tetra-ubiquitin. Binding kinetics were obtained from fitting processed data to a 1:1 binding model. Graphs show representative BLI sensorgram and fit for TNK1-UBA/TAB2-UBD and tetra-ubiquitin.
c. Mock- or TNK1-expressing Ba/F3 cells from Fig. 3g were immunoblotted for global phospho-tyrosine as an indicator of TNK1 activity. Cells were also immunoblotted for GFP (expressed from the TNK1-expressing retrovirus) as a loading control. Blot shows a representative image from 3 biological replicates.
d. HEK293T cells stably expressing HA-14-3-3 were transfected with WT FLAG-TNK1, followed by immunoprecipitation on HA resin and immunoblotting for FLAG. Blot shows a representative image from 3 biological replicates.
e. Quantitation of Fig. 3g in the form of transformation rate, as well as panel c and d from n=3 replicates, shown with average signals normalized to WT TNK1. Error bars represent SEM.
f. HEK-293A cells expressing TNK1-GFP (WT or AAA) were analyzed by confocal imaging for co-localization with ubiquitin. Images were analyzed using Huygens Essential software. Pearson's coefficient was used to measure co-localization. Graph show average Pearson's coefficient with error bars representing SEM. (n=20).
g. HEK-293T cells expressing WT FLAG-TNK1 were treated with vehicle (DMSO) or 10 μM MG132 for 2 hours and biochemically fractionated into cytosol and heavy membrane fractions. Immunoblotting for FLAG-TNK1 was followed. Fractions were also immunoblotting for VDAC and actin as loading controls. Graph shows relative HM/C ratio normalized to vehicle. Quantitation is from multiple biological replicates (n=5) with error bars representing SEM.

a

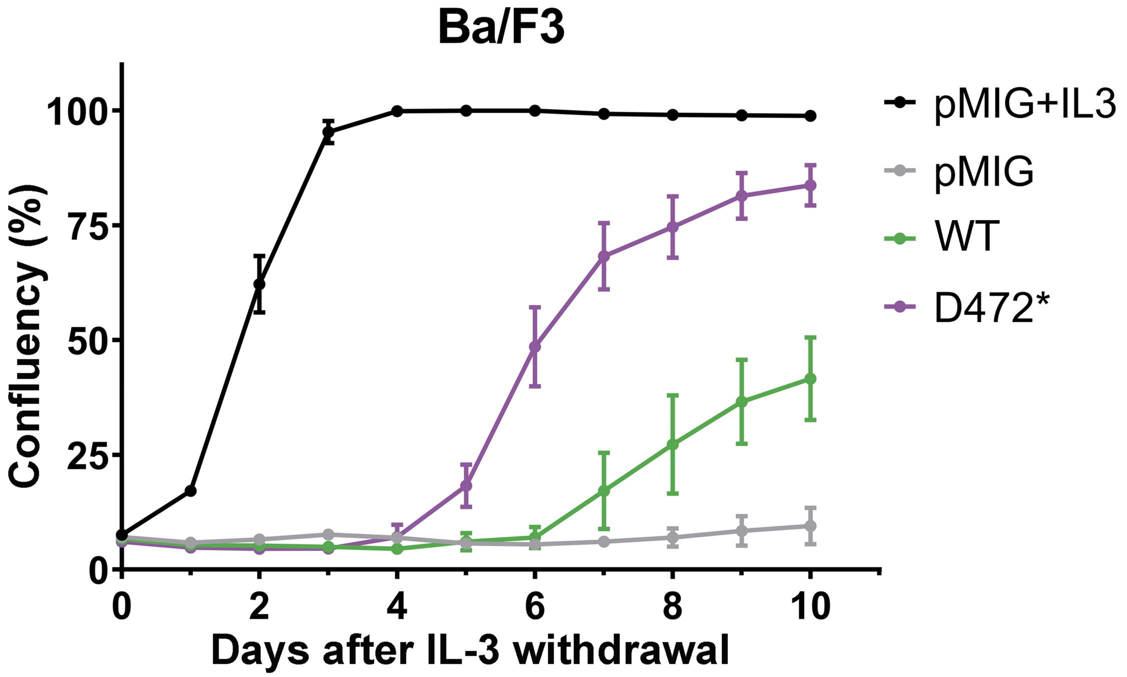

b

Status	Effect	Activity
WT<br>14-3-3 binding; Ubiquitin binding<br>H_2N – SAM – Kinase Domain – SH3 – Proline-rich – UBA – COOH	NA	- +
AAA<br>14-3-3 binding (X); Ubiquitin binding<br>H_2N – … – COOH<br>* SA mutant	No 14-3-3 binding	+ + +
ΔUBA<br>14-3-3 binding; Truncated at UBA<br>H_2N – …	No ubiquitin binding	- -
ΔUBA-AAA<br>14-3-3 binding (X); Truncated at UBA<br>H_2N – …<br>* SA mutant	No 14-3-3 binding<br>No ubiquitin binding	- + +
Paracentric inversion, HL line<br>Hodgkin lymphoma inversion truncates TNK1 at D472<br>H_2N – …	No 14-3-3 binding<br>No ubiquitin binding	- + +

Supplementary Figure 5. A naturally occurring mutant of TNK1 from Hodgkin Lymphoma activates TNK1 via truncation of the 14-3-3 binding site.

a. Ba/F3 cells were transduced with either pMIG-empty vector, WT TNK1 or the D472* TNK1 (to mimic the naturally occurring mutation in L540 cells) were analyzed for IL-3 independent growth as in Fig. 3g. Graph represents mean cell confluency from 3 biological replicates. Error bars represent SEM.
b. A table showing the effect of various mutations on TNK1 kinase activity.

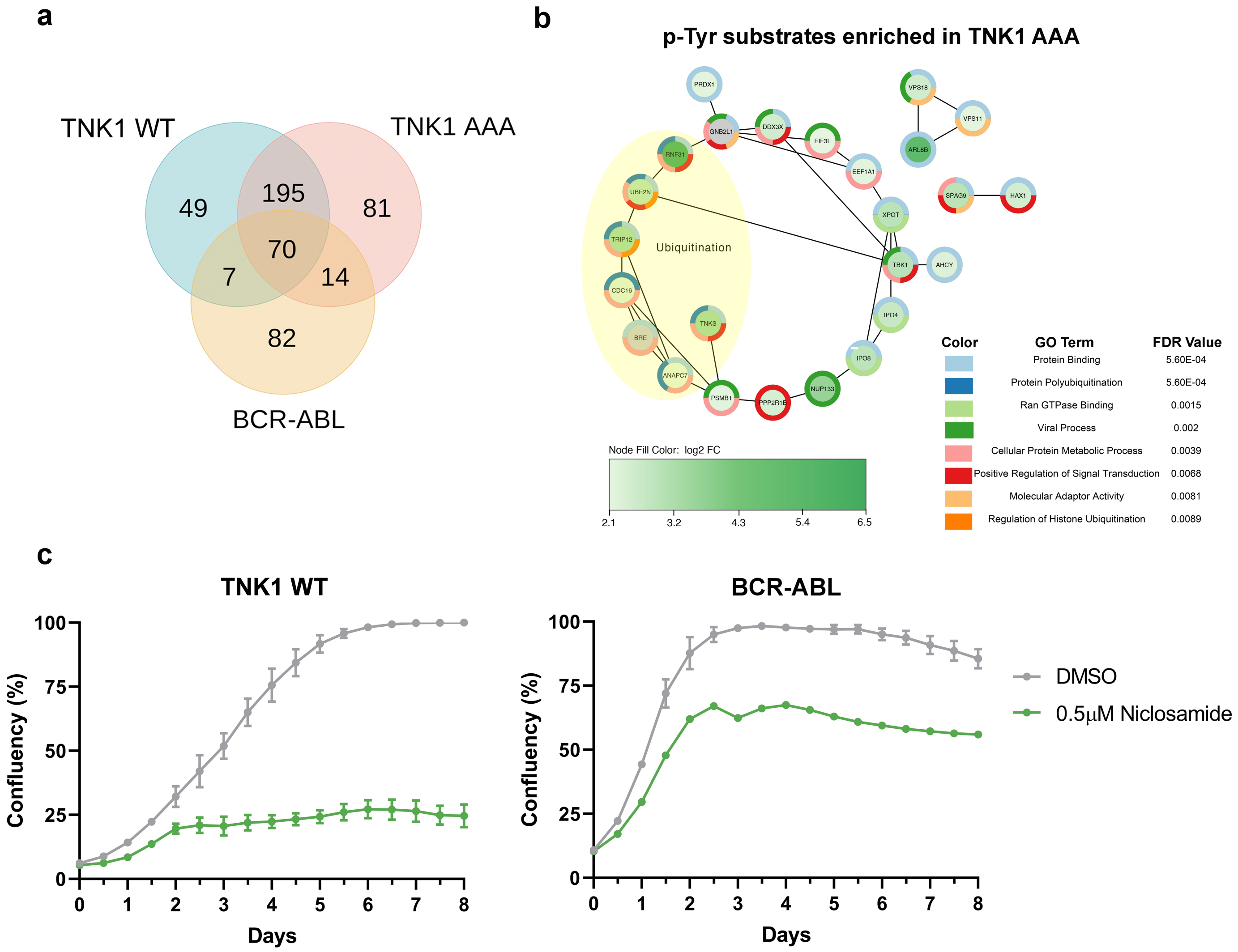

Supplementary Figure 6. Analysis of putative TNK1 phospho-substrates reveals pro-growth signaling through STAT3.

a. Venn diagram of unique proteins identified through phospho-tyrosine proteomics of TNK1- and BCR-ABL-expressing cells. Included proteins are at least 2-fold higher in signal comparative to background (MOCK) and have a p-value lower than 0.05.

b. Phospho-tyrosine substrates that were significant and displayed a fold change of two or greater (relative to control) in TNK1-AAA alone were subjected to STRING DB network analysis and visualized in Cytoscape. Node color represents magnitude of fold change. A ubiquitin-related subset of interactors (highlighted) was selected using GO-terms and manual curation.

c. TNK1 WT-driven and BCR-ABL-driven Ba/F3 cells were treated with 0.5µM Niclosamide. Mean cell confluency from 3 biological replicates were analyzed as in Fig. 3g. Error bars represent SEM.

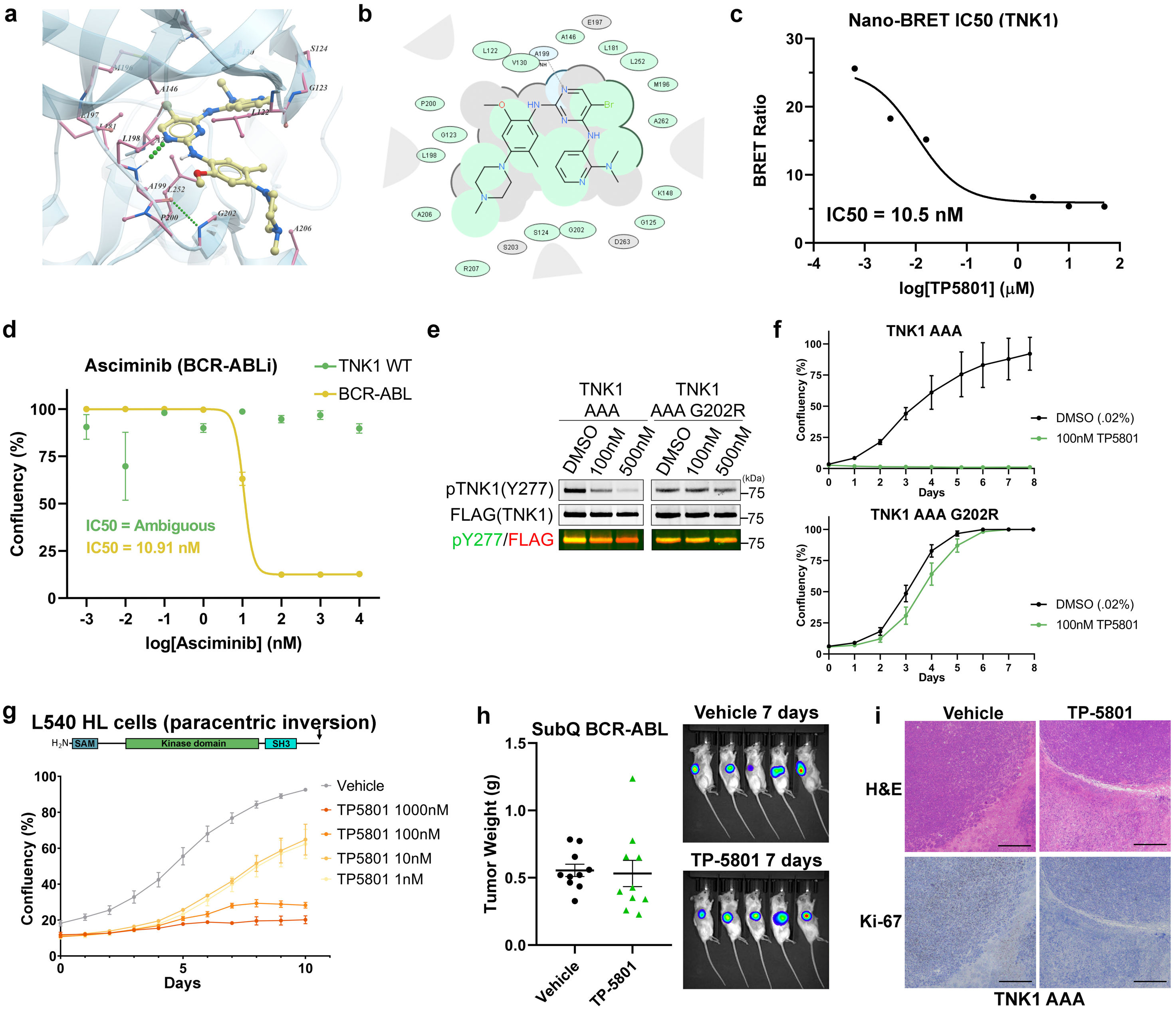
a
S124
A146
G123
E197
L181
L198
L122
A199
L252
P200
G202
A206
b
E197
A146
L122
A199
V130
L181
L252
M196
P200
G123
A262
L198
K148
A206
G125
S124
G202
S203
D263
R207
c
Nano-BRET IC50 (TNK1)
BRET Ratio
IC50 = 10.5 nM
log[TP5801] (µM)
d
Asciminib (BCR-ABLi)
TNK1 WT
BCR-ABL
Confluency (%)
IC50 = Ambiguous
IC50 = 10.91 nM
log[Asciminib] (nM)
e
TNK1 AAA
TNK1 AAA G202R
DMSO
100nM
500nM
(kDa)
pTNK1(Y277)
FLAG(TNK1)
pY277/FLAG
75
f
TNK1 AAA
Confluency (%)
Days
DMSO (.02%)
100nM TP5801
TNK1 AAA G202R
g
L540 HL cells (paracentric inversion)
H2N-
SAM
Kinase domain
SH3
Confluency (%)
Days
Vehicle
TP5801 1000nM
TP5801 100nM
TP5801 10nM
TP5801 1nM
h
SubQ BCR-ABL
Tumor Weight (g)
Vehicle
TP-5801
Vehicle 7 days
TP-5801 7 days
i
Vehicle
TP-5801
H&E
Ki-67
TNK1 AAA

Supplementary Figure 7. The development of a potent TNK1 inhibitor, TP-5801.

a. Image shows the TP-5801 docking pose in the TNK1 active site homology model.
b. 2D interactome of docking simulation of TP-5801 in the TNK1 active site homology model.
c. IC50 graph of TP5801 against TNK1, as determined by NanoBRETTM analysis. Graph shows average BRET Ratio from 2 replicates.
d. IC50 graph of TNK1-WT driven Ba/F3 cells or BCR-ABL-driven Ba/F3 cells treated with Asciminib (1pM to 10mM) for 72 hours. Cell confluency was measured using IncuCyte imaging system. Graphs shows average cell confluency from 3 biological replicates. Error bars represent SEM.
e. HEK293T cells expressing FLAG-TNK1 AAA or FLAG-TNK1-AAA-G202R were treated vehicle or indicated concentration of TP-5801 for 4 hours. FLAG-TNK1 was immunoprecipitated on FLAG resin and immunoblotted for TNK1 pY277. Blot shows representative image from 3 biological replicates.
f. Ba/F3 cells transduced with TNK1-AAA or TNK1-AAA-G202R were maintained without IL-3. Cells treated with vehicle (DMSO) or 100nM TP-5801 and growth was monitored by Incucyte live cell imaging. Graph shows that average cell confluency with error bars representing SEM from 3 biological replicates.
g. Schematic diagram showing the truncated from of TNK1 as a result of paracentric inversion in L540 cells. L540 cells were treated with the indicated doses of TP-5801 or vehicle (DMSO) and analyzed for cell growth in an Incucyte live-cell growth chamber. Error bars represent SEM from three replicates.
h. NOD/SCID mice were subcutaneously injected with BCR-ABL driven Ba/F3-luc cells. Once tumor size reached 150-250mm3, mice (n=10 for each group) were treated once daily by oral gavage with either vehicle or 50mg/kg TP-5801 for seven days. Luminescent signal was imaged and quantified using IVIS imaging. Box plot shows median, first and third quartile of tumor weight. Images show the representative luminescent signal of the mice from each group.
i. Formalin fixed and paraffin-embedded tumor tissue from Fig.6e (TNK1-AAA mice) were immunostained for the proliferative nuclear antigen Ki-67 and counterstained with hematoxylin and eosin (H&E). Figure shows representative images from multiple tumors (vehicle n=11; TP-5801 n=12). Scale bar = 250µm.

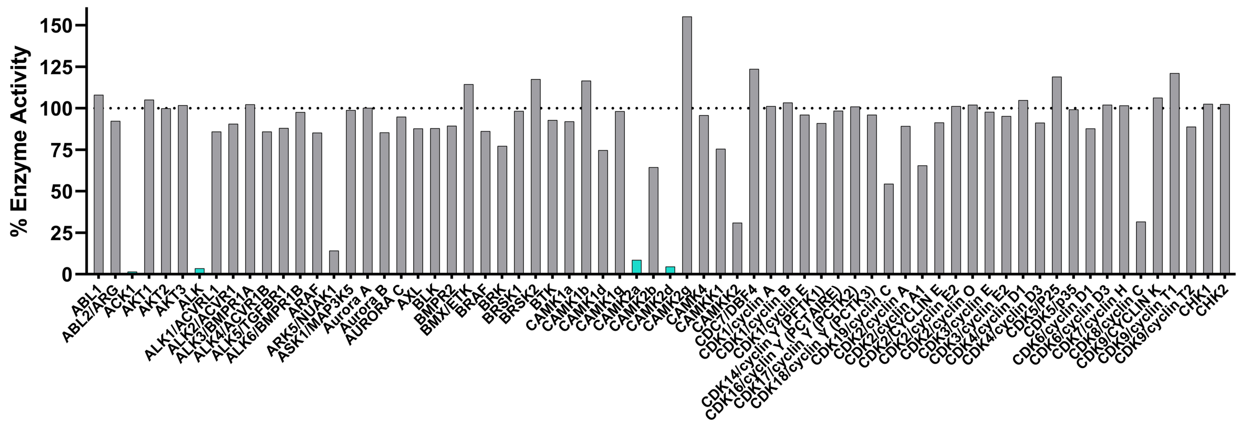
% Enzyme Activity
0
25
50
75
100
125
150
ABL1
ABL2/ARG
ACK1
AKT1
AKT2
AKT3
ALK
ALK1/ACVRL1
ALK2/ACVR1
ALK3/BMPR1A
ALK4/ACVR1B
ALK5/TGFBR1
ALK6/BMPR1B
ARAF
ARK5/NUAK1
ASK1/MAP3K5
Aurora A
Aurora B
AURORA C
AXL
BLK
BMPR2
BMX/ETK
BRAF
BRK
BRSK1
BRSK2
BTK
CAMK1a
CAMK1b
CAMK1d
CAMK1g
CAMK2a
CAMK2b
CAMK2d
CAMK2g
CAMK4
CAMKK1
CAMKK2
CDC7/DBF4
CDK1/cyclin A
CDK1/cyclin B
CDK1/cyclin E
CDK14/cyclin Y (PFTAIRE)
CDK16/cyclin Y (PCTK1)
CDK17/cyclin Y (PCTK2)
CDK18/cyclin Y (PCTK3)
CDK19/cyclin C
CDK2/cyclin A
CDK2/cyclin A1
CDK2/CYCLIN E
CDK2/cyclin E2
CDK2/cyclin O
CDK3/cyclin E
CDK3/cyclin E2
CDK4/cyclin D1
CDK4/cyclin D3
CDK5/P25
CDK5/p35
CDK6/cyclin D1
CDK6/cyclin D3
CDK7/cyclin H
CDK8/CYCLIN C
CDK9/CYCLIN K
CDK9/cyclin T1
CDK9/cyclin T2
CHK1
CHK2

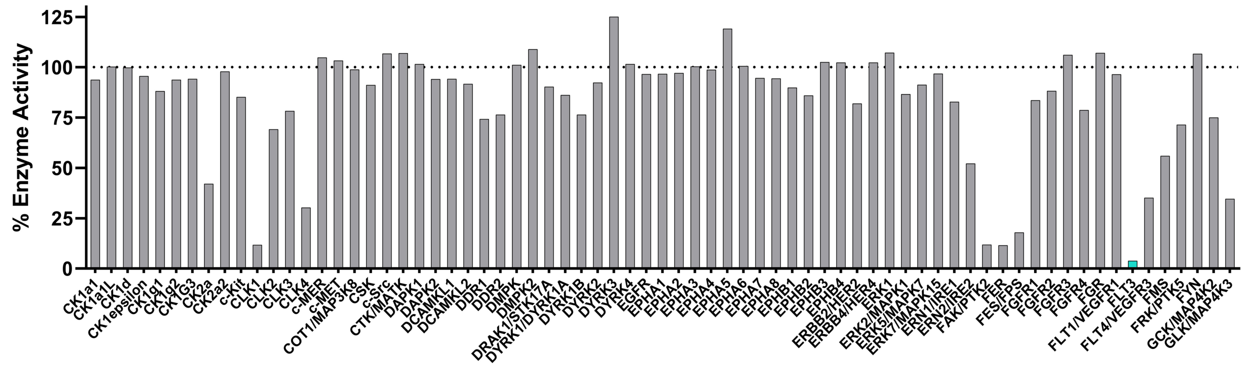
% Enzyme Activity
0
25
50
75
100
125
CK1a1
CK1a1L
CK1d
CK1epsilon
CK1g1
CK1g2
CK1G3
CK2a
CK2a2
c-Kit
CLK1
CLK2
CLK3
CLK4
c-MER
c-MET
COT1/MAP3K8
CSK
c-Src
CTK/MATK
DAPK1
DAPK2
DCAMKL1
DCAMKL2
DDR1
DDR2
DMPK
DNAPK
DRAK1/STK17A
DYRK1/DYRK1A
DYRK1B
DYRK2
DYRK3
DYRK4
EGFR
EPHA1
EPHA2
EPHA3
EPHA4
EPHA5
EPHA6
EPHA7
EPHA8
EPHB1
EPHB2
EPHB3
EPHB4
ERBB2/HER2
ERBB4/HER4
ERK1
ERK2/MAPK1
ERK5/MAPK7
ERK7/MAPK15
ERN1/IRE1
ERN2/IRE2
FAK/PTK2
FER
FES/FPS
FGFR1
FGFR2
FGFR3
FGFR4
FGR
FLT1/VEGFR1
FLT3
FLT4/VEGFR3
FMS
FRK/PTK5
FYN
GCK/MAP4K2
GLK/MAP4K3

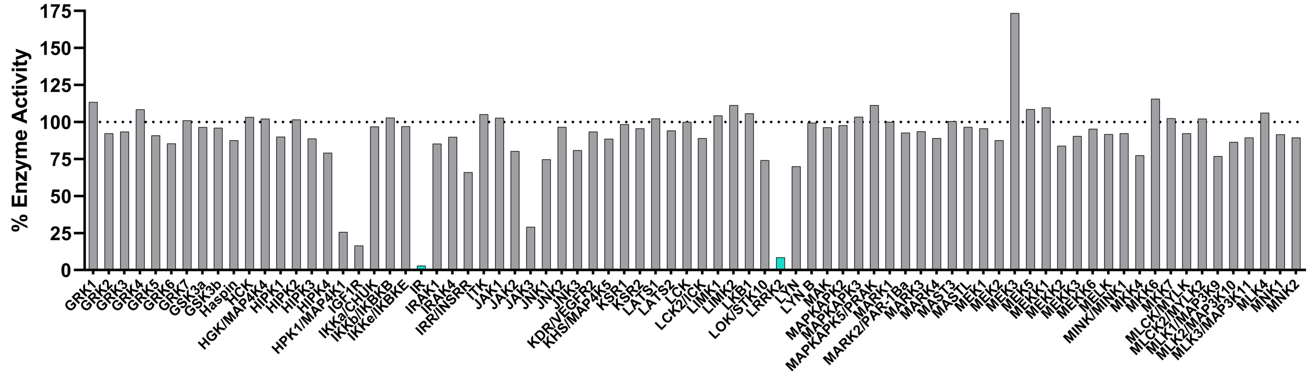
% Enzyme Activity
0
25
50
75
100
125
150
175
GRK1
GRK2
GRK3
GRK4
GRK5
GRK6
GRK7
GSK3a
GSK3b
Haspin
HCK
HGK/MAP4K4
HIPK1
HIPK2
HIPK3
HIPK4
HPK1/MAP4K1
IGF1R
IKKa/CHUK
IKKb/IKBKB
IKKe/IKBKE
IR
IRAK1
IRAK4
IRR/INSRR
ITK
JAK1
JAK2
JAK3
JNK1
JNK2
JNK3
KDR/VEGFR2
KHS/MAP4K5
KSR1
KSR2
LATS1
LATS2
LCK
LCK2/ICK
LIMK1
LIMK2
LKB1
LOK/STK10
LRRK2
LYN
LYN B
MAK
MAPKAPK2
MAPKAPK3
MAPKAPK5/PRAK
MARK1
MARK2/PAR-1Ba
MARK3
MARK4
MAST3
MASTL
MEK1
MEK2
MEK3
MEK5
MEKK1
MEKK2
MEKK3
MEKK6
MELK
MINK/MINK1
MKK4
MKK6
MKK7
MLCK/MYLK
MLCK2/MYLK2
MLK1/MAP3K9
MLK2/MAP3K10
MLK3/MAP3K11
MLK4
MNK1
MNK2

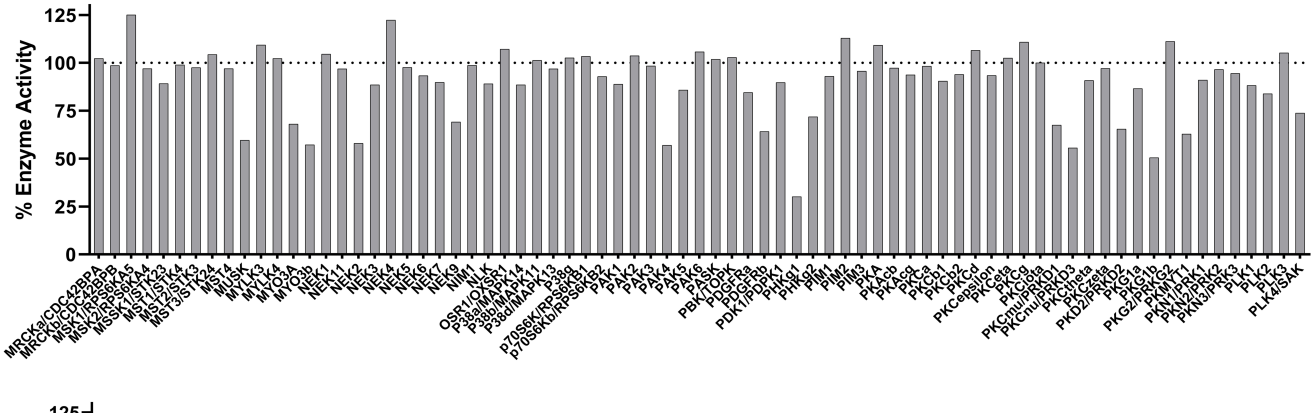
% Enzyme Activity
0
25
50
75
100
125
MRCKa/CDC42BPA
MRCKb/CDC42BPB
MSK1/RPS6KA5
MSK2/RPS6KA4
MSSK1/STK23
MST1/STK4
MST2/STK3
MST3/STK24
MST4
MUSK
MYLK3
MYLK4
MYO3a
MYO3b
NEK1
NEK11
NEK2
NEK3
NEK4
NEK5
NEK6
NEK7
NEK9
NIM1
NLK
OSR1/OXSR1
P38a/MAPK14
P38b/MAPK11
P38d/MAPK13
P38g
p70S6K/RPS6KB1
p70S6Kb/RPS6KB2
PAK1
PAK2
PAK3
PAK4
PAK5
PAK6
PASK
PBK/TOPK
PDGFRa
PDGFRb
PDK1/PDPK1
PHKg1
PHKg2
PIM1
PIM2
PIM3
PKA
PKAcb
PKAcg
PKCa
PKCb1
PKCb2
PKCd
PKCepsilon
PKCeta
PKCg
PKCiota
PKCmu/PRKD1
PKCnu/PRKD3
PKCtheta
PKCzeta
PKD2/PRKD2
PKG1a
PKG1b
PKG2/PRKG2
PKMYT1
PKN1/PRK1
PKN2/PRK2
PKN3/PRK3
PLK1
PLK2
PLK3
PLK4/SAK

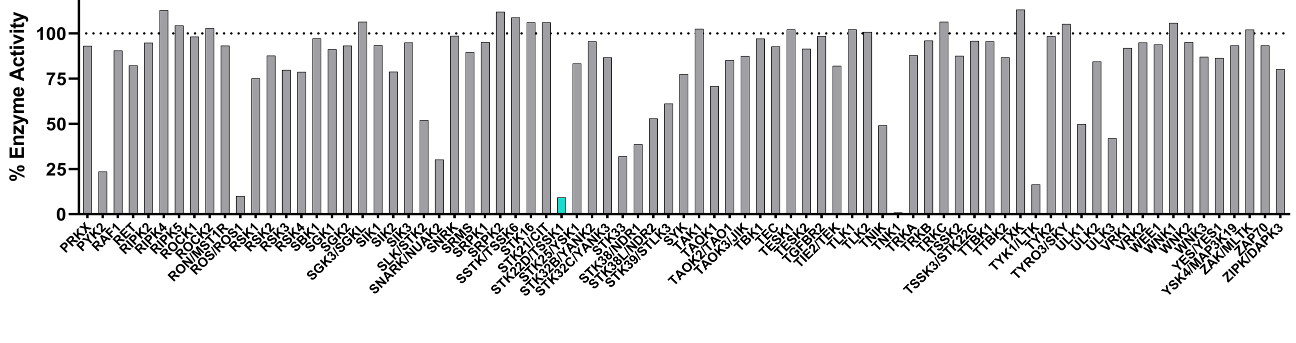
% Enzyme Activity
0
25
50
75
100
125
PRKX
PYK2
RAF1
RET
RIPK2
RIPK4
RIPK5
ROCK1
ROCK2
RON/MST1R
ROS/ROS1
RSK1
RSK2
RSK3
RSK4
SBK1
SGK1
SGK2
SGK3/SGKL
SIK1
SIK2
SIK3
SLK/STK2
SNARK/NUAK2
SNRK
SRMS
SRPK1
SRPK2
SSTK/TSSK6
STK16
STK22D/TSSK1
STK25/YSK1
STK32B/YANK2
STK32C/YANK3
STK33
STK38/NDR1
STK38L/NDR2
STK39/STLK3
SYK
TAK1
TAOK1
TAOK2/TAO1
TAOK3/JIK
TBK1
TEC
TESK1
TGFBR2
TIE2/TEK
TLK1
TLK2
TNIK
TNK1
TRKA
TRKB
TRKC
TSSK2
TSSK3/STK22C
TTBK1
TTBK2
TXK
TYK1/LTK
TYK2
TYRO3/SKY
ULK1
ULK2
ULK3
VRK1
VRK2
WEE1
WNK1
WNK2
WNK3
YES/YES1
YSK4/MAP3K19
ZAK/MLTK
ZAP70
ZIPK/DAPK3

Supplementary Figure 8. In vitro kinase panel screen with TP-5801

TP-5801 (0.3 μM) was tested in duplicate against 371 kinases. Control compounds (in the supplementary data set) were tested in serial dilutions to establish the lower boundary of the assay. Average percent enzyme activity is shown from two replicates. Cyan bars represent inhibition >90%.

Supplementary Table 1. List of primers used for mutagenesis and RT-qPCR

Application	Primer Name	Sequence
Mutagenesis	TNK1-aa384Truncation-F	CGGAAGCATGaTGTGTGAGGGATGTC
Mutagenesis	TNK1-aa384Truncation-R	AAGGCCCGGCCTCTTGCA
Mutagenesis	TNK1-aa450Truncation-F	TCCAGTCCACtGAGGCACCCCTG
Mutagenesis	TNK1-aa450Truncation-R	CGGGTGGCTGGCAAGCCC
Mutagenesis	TNK1-aa495Truncation-F	GGAGAGGATGtAAGGCATTTCCAG
Mutagenesis	TNK1-aa495Truncation-R	AGGGGCATGTTCCTCCTC
Mutagenesis	TNK1-aa585Truncation-F	GGAGGCCTCTaGTCCGATCCTG
Mutagenesis	TNK1-aa585Truncation-R	AGAGAGGGCAGCGGCTTT
Mutagenesis	TNK1-S500A-F	GAAAGGCATTgCCAGGAGTCTG
Mutagenesis	TNK1-S500A-R	ATCCTCTCCAGGGGCATG
Mutagenesis	TNK1-S502A-F	CATTTCCAGGgcTCTGGAGTCAGTTCTG
Mutagenesis	TNK1-S502A-R	CCTTTCATCCTCTCCAGG
Mutagenesis	TNK1-S505A-F	GAGTCTGGAGgCAGTTCTGTC
Mutagenesis	TNK1-S505A-R	CTGGAAATGCCTTTCATCC
Mutagenesis	TNK1-AAA-F	CTCTGGAGgCAGTTCTGTCCC
Mutagenesis	TNK1-AAA-R	CCCTGGCAATGCCTTTCATCC
Mutagenesis	TNK1-EEE-F	CTGGAGCTCGGTCCTCGTCCCACA
Mutagenesis	TNK1-EEE-R	AACTTCCTCCAGACTCCTGGAAATGC
Mutagenesis	TNK1-S68A-F	AAGGCTACGTgCTGGGCCTAA
Mutagenesis	TNK1-S68A-R	TTCAGAGCTTCGGACAGTC
Mutagenesis	TNK1-T91A-F	CAAGGAGCCCgCCCTGCCCTC
Mutagenesis	TNK1-T91A-R	TGCTCAGGGGCAAAACCTCC
Mutagenesis	TNK1-T392A-F	GAGGGATGTCgCAGAACCAGG
Mutagenesis	TNK1-T392A-R	ACACAACATGCTTCCGAAG
Mutagenesis	TNK1-S434A-F	CAAAGTGGGCgccTTCCCAGCCTC
Mutagenesis	TNK1-S434A-R	AAGGTGCGACCATTCTGG
Mutagenesis	TNK1-K148R-F	GTGGCTGTCAgGTCCCTCCGG
Mutagenesis	TNK1-K148R-R	TGGGACACTCTTGCCACTG
Mutagenesis	TNK1-UBA-MUT-M596D-F	GAGGAAGATTGATGAGGTGGAGCTGAGTGTGC
Mutagenesis	TNK1-UBA-MUT-M596D-R	TGCAACTCAGGATCGGAC
Mutagenesis	TNK1-UBA-MUT-L600D-F	GGAGGTGGAGGATAGTGTGCATG
Mutagenesis	TNK1-UBA-MUT-L600D-R	ATAATCTTCCTCTGCAAC
Mutagenesis	TNK1-UBA-MUT-I625D-F	GGTTTCTGCCGACCGGAACCTCAAG
Mutagenesis	TNK1-UBA-MUT-I625D-R	ACATCTCCCCCAGTGGCT
Mutagenesis	TNK1-UBA-MUT-F634D-F	AGATCAGCTCGACCACCTGAGTAG
Mutagenesis	TNK1-UBA-MUT-F634D-R	ACCTTGAGGTTCCGGATG
Mutagenesis	TNK1-UBA-MUT-L636D-F	GCTCTTCCACGATAGTAGCCGGTC
Mutagenesis	TNK1-UBA-MUT-L636D-R	TGATCTACCTTGAGGTTC
Mutagenesis	TNK1-G202R-F	GGCGCCACTGcGCTCCCTGCA
Mutagenesis	TNK1-G202R-R	AGCTCCATCACCATCTGCAGAG
RT-qPCR	GAPDH-F	CCCCATGTTCGTCATGGGTGTG

RT-qPCR	GAPDH-R	GGCATGGACTGTGGTCATGAGTC
RT-qPCR	MARK1-F	TGGATATACTGAACCACACATCCAGC
RT-qPCR	MARK1-R	TATTTTCACAGCAACCTCTCTACCAG
RT-qPCR	MARK2-F	AGTTATCCTCTATACACTGGTCAGCG
RT-qPCR	MARK2-R	GATTTGCTCTAAAGTGCCTCTCTTGC
