# Supplementary material for: TNK1 is a ubiquitin-binding and 14-3-3-regulated kinase that can be targeted to block tumor growth
Source: Nat Commun. 2021 Sep 9;12:5337. doi: 10.1038/s41467-021-25622-3 (PMC8429728; doi:10.1038/s41467-021-25622-3)
Supplement: Supplementary file 3 — Description of Additional Supplementary Files [file 41467_2021_25622_MOESM3_ESM.pdf]

Description of Additional Supplementary Files

File Name: Supplementary Data 1
Description: Raw data from radiometric kinase assay screen with 245 individual S/T protein kinases from Supplementary Figure 3d.

File Name: Supplementary Data 2
Description: In vitro kinase screen with TP-5801 from Supplementary Figure 8.
