# Supplementary material for: TNK1 is a ubiquitin-binding and 14-3-3-regulated kinase that can be targeted to block tumor growth
Source: Nat Commun. 2021 Sep 9;12:5337. doi: 10.1038/s41467-021-25622-3 (PMC8429728; doi:10.1038/s41467-021-25622-3)
Supplement: Supplementary file 6 — Reporting Summary [file 41467_2021_25622_MOESM6_ESM.pdf]

nature research

Corresponding author(s): Joshua L. Andersen

Last updated by author(s): Apr 20, 2021

Reporting Summary

Nature Research wishes to improve the reproducibility of the work that we publish. This form provides structure for consistency and transparency in reporting. For further information on Nature Research policies, see our Editorial Policies and the Editorial Policy Checklist.

Statistics

For all statistical analyses, confirm that the following items are present in the figure legend, table legend, main text, or Methods section.

n/a	Confirmed	
☐	☒	The exact sample size (*n*) for each experimental group/condition, given as a discrete number and unit of measurement
☐	☒	A statement on whether measurements were taken from distinct samples or whether the same sample was measured repeatedly
☐	☒	The statistical test(s) used AND whether they are one- or two-sided<br>*Only common tests should be described solely by name; describe more complex techniques in the Methods section.*
☒	☐	A description of all covariates tested
☒	☐	A description of any assumptions or corrections, such as tests of normality and adjustment for multiple comparisons
☐	☒	A full description of the statistical parameters including central tendency (e.g. means) or other basic estimates (e.g. regression coefficient) AND variation (e.g. standard deviation) or associated estimates of uncertainty (e.g. confidence intervals)
☐	☒	For null hypothesis testing, the test statistic (e.g. *F*, *t*, *r*) with confidence intervals, effect sizes, degrees of freedom and *P* value noted<br>*Give P values as exact values whenever suitable.*
☒	☐	For Bayesian analysis, information on the choice of priors and Markov chain Monte Carlo settings
☒	☐	For hierarchical and complex designs, identification of the appropriate level for tests and full reporting of outcomes
☐	☒	Estimates of effect sizes (e.g. Cohen's *d*, Pearson's *r*), indicating how they were calculated

Our web collection on statistics for biologists contains articles on many of the points above.

Software and code

Policy information about availability of computer code

Data collection	No custom code was used for data collection. Scaffold 5 and PEAKS 10.6 were used for mass spectrometry data. Li-Cor Image Studio 5.0 was used for detection of immunoblot signals. Perkin Elmer Living Image software version 4.7.3 was used for detection of in vivo IVIS luciferase signal. Leica Application Suite X software version 3.1.1.15751 was used to collect confocal images.
Data analysis	PRISM GraphPad 9.0 was used for statistical analysis of data and generation of figure graphs. Hyugens deconvolution and 3D analysis software 19.04.0p6 64b was used for quantitation and analysis of confocal imaging

For manuscripts utilizing custom algorithms or software that are central to the research but not yet described in published literature, software must be made available to editors and reviewers. We strongly encourage code deposition in a community repository (e.g. GitHub). See the Nature Research guidelines for submitting code & software for further information.

Data

Policy information about availability of data

All manuscripts must include a data availability statement. This statement should provide the following information, where applicable:

- Accession codes, unique identifiers, or web links for publicly available datasets
- A list of figures that have associated raw data
- A description of any restrictions on data availability

The TNK1 BioID and phospho-tyrosine proteomics data have been deposited in the MassIVE database (massive.ucsd.edu) under accession codes MSV000087618 and MSV000087623, respectively. All other relevant data supporting key findings of this study are available in the article or in supplementary data files or from the corresponding author upon reasonable request. Source data are included in this paper.

Field-specific reporting

Please select the one below that is the best fit for your research. If you are not sure, read the appropriate sections before making your selection.

☒ Life sciences ☐ Behavioural & social sciences ☐ Ecological, evolutionary & environmental sciences

For a reference copy of the document with all sections, see nature.com/documents/nr-reporting-summary-flat.pdf

Life sciences study design

All studies must disclose on these points even when the disclosure is negative.

Sample size	For mouse studies, sample size was estimated via power analysis. For all other experiments, no sample size calculation was done. Instead, sample sizes were estimated based on pilot studies in our group. For all western blot experiments except figure S2F, multiple biological replicates were used to determine statistically significant differences between experiments. For figure S2F, we only performed one experiment because it was a confirmation that 14-3-3 binds to TNK1 in a phospho-dependent manner, which we went on to show through a variety of other experiments. For confocal imaging analysis of diffuse versus punctate TNk1 localization, we counted 50 individual cells per experiment, which we reasoned was sufficient to show a qualitative trend in subcellular distribution. For confocal imaging of TNK1 puncta volume and colocalization , a sufficient number of replicates were analyzed by software to determine significant differences between experiments. For phospho-tyrosine proteomics and biolayer inferometry, we chose 3 replicates per experiment because it was the minimum number needed to calculate significant differences between experiments.
Data exclusions	No data were excluded
Replication	Western blot experiments were repeated with at least 3 biological replicates, with the exception of figure S2F (co-IP of 14-3-3 K49Q mutant), which was only done once . All other experiments in this manuscript were repeated with sufficient replicates to demonstrate statistical significance. The number of replicates in each experiment is indicated in the corresponding figure legend
Randomization	For animal studies, mice were allocated randomly into the individual groups, with the exception of gender wherein efforts were made to balance male and female representation in each group. For biochemical/molecular experiments, no randomization was necessary given the nature of the experiments.
Blinding	The analysis of diffuse versus punctate localization of TNK1 in figure 1B was performed in a blinded manner wherein the person doing the analysis was not aware of experimental grouping. Subsequent analysis of diffuse versus punctate TNK1 localization was not blinded. No blinding was used for other experiments.

Reporting for specific materials, systems and methods

We require information from authors about some types of materials, experimental systems and methods used in many studies. Here, indicate whether each material, system or method listed is relevant to your study. If you are not sure if a list item applies to your research, read the appropriate section before selecting a response.

Materials & experimental systems

n/a	Involved in the study	
☐	☒	Antibodies
☐	☒	Eukaryotic cell lines
☒	☐	Palaeontology and archaeology
☐	☒	Animals and other organisms
☐	☒	Human research participants
☐	☒	Clinical data
☒	☐	Dual use research of concern

Methods

n/a	Involved in the study	
☒	☐	ChIP-seq
☒	☐	Flow cytometry
☒	☐	MRI-based neuroimaging

Antibodies

Antibodies used	Antibody name, company, catalog number, dilution used<br><br>Anti-TNK1 pS502 phospho-specific antibody, custom made/ordered from Pacific Immunology, 1:100<br>Phospho-TNK1 (Tyr277) (D46E7) Rabbit mAb Cell Signaling Technology 5638S, 1:1000<br>Monoclonal ANTI-FLAG® M2 antibody Sigma-Aldrich F1804, 1:1000<br>DYKDDDDK Tag (9A3) Mouse mAb Cell Signaling Technology 8146S, 1:1000<br>14-3-3 Pan Polyclonal Antibody Thermo Fisher Scientific 510700, 1:1000<br>14-3-3 zeta antibody GeneTex GTX101075, 1:1000<br>HA-Tag(6E2) Mouse Antibody Cell Signaling Technology 2367S, 1:1000<br>Phospho Tyrosine (P Tyr 1000) MultiMab™ Rabbit mAb Cell Signaling Technology 8954s, 1:1000<br>Ubiquitin Antibody Rabbit Cell Signaling Technology 3933S,1:1000

Ubiquitin (P4D1) Mouse mAb Cell Signaling Technology 3936S, 1:1000
VU-1 antibody (ubiquitin) LifeSensors vu101, 1:1000
MARK1 Rabbit Polyclonal Antibody Proteintech Group 21552-1-AP, 1:500
MARK2 Rabbit Polyclonal Antibody Proteintech Group 15492-1-AP, 1:500
MARK3 Cell Signaling Technology 9311S, 1:500
MARK4 MyBioSource MBS8208929, 1:500
GST (26H1) Mouse mAb Cell Signaling Technology 2624S, 1:1000
STAT1 Cell Signaling Technology 14994, 1:1000
Phospho-Stat3 (Tyr705) (M9C6) Mouse mAb Cell Signaling Technology 4113, 1:1000
Stat3 (D3Z2G) Rabbit mAb Cell Signaling Technology 12640, 1:1000
PhosphoSTAT5 (Tyr694) Cell Signaling Technology 4322, 1:1000
STAT5 Cell Signaling Technology 25656, 1:1000
GFP (4B10) Mouse mAb Cell Signaling Technology 2955S, 1:1000,
IRDye® 800CW Goat anti-Rabbit IgG (H + L) Li-cor 92632211, 1:10,000
IRDye® 680RD Goat anti-Mouse IgG (H + L) Li-cor 926-68070, 1:10,000
Goat anti-Mouse IgG1 Cross-adsorbed Secondary Antibody, Alex Fluor 633 Invitrogen A-21126, 1:500
Ubiquitin antibody (anti-mouse), Cell Signaling Technology, 3936, 1:1000

Validation

Antibody; Reactivity; Tested applications
Anti-TNK1 pS502 antibody; Rab, H; WB
Phospho-TNK1 (Tyr277) (D46E7) Rabbit mAb Cell Signaling Technology 5638S; H; WB
Monoclonal ANTI-FLAG® M2 antibody Sigma-Aldrich F1804; not applicable (tag); IHC, IF, WB
DYKDDDDK Tag (9A3) Mouse mAb Cell Signaling Technology 8146S; not applicable (tag); WB, IP, IHC, IF, F
14-3-3 Pan Polyclonal Antibody Thermo Fisher Scientific 510700; Bov, C ele, H, M, R, Xen; WB, IHC, Elisa, IF
14-3-3 zeta antibody GeneTex GTX101075; H, M; WB, IF, IHC
HA-Tag(6E2) Mouse Antibody Cell Signaling Technology 2367S; not applicable (tag); WB, IHC, IF, F
Phospho Tyrosine (P Tyr 1000) MultiMab™ Rabbit mAb Cell Signaling Technology 8954s; All; WB, IP, IF, F
Ubiquitin Antibody Rabbit Cell Signaling Technology 3933S; All; WB, IHC
Ubiquitin (P4D1) Mouse mAb Cell Signaling Technology 3936S; All; WB, IHC
VU-1 antibody (ubiquitin) LifeSensors vu101; All; IHC, WB, IF
MARK1 Rabbit Polyclonal Antibody Proteintech Group 21552-1-AP; H, M, R; WB, IP, IHC, Elisa
MARK2 Rabbit Polyclonal Antibody Proteintech Group 15492-1-AP; H, M, R; WB, IP, IHC, Elisa
MARK3 Cell Signaling Technology 9311S; H, M, R; WB, IP
MARK4 MyBioSource MBS8208929; H, M, R; WB, IP, IHC, IF, ICC
GST (26H1) Mouse mAb Cell Signaling Technology 2624S; All; WB, IP, IF
STAT1 Cell Signaling Technology 14994; H, M, R, Mk; WB, IP, IF, F, ChIP, IHC
Phospho-Stat3 (Tyr705) (M9C6) Mouse mAb Cell Signaling Technology 4113; H, M, R, Mk; WB, IP, IHC, IF, F
Stat3 (D3Z2G) Rabbit mAb Cell Signaling Technology 12640; H, M, R, Mk; WB, IP, IHC, IF, F
PhosphoSTAT5 (Tyr694) Cell Signaling Technology; H, M; WB, IF, F
STAT5 Cell Signaling Technology 25656; H; WB, IP, IHC, IF
GFP (4B10) Mouse mAb Cell Signaling Technology 2955S; All; WB, IHC, F
IRDye® 800CW Goat anti-Rabbit IgG (H + L) Li-cor 92632211; Rab; WB, IHC, IF,
IRDye® 680RD Goat anti-Mouse IgG (H + L) Li-cor 926-68070; M; WB, IHC, IF
Goat anti-Mouse IgG1 Alex Fluor 633 Invitrogen A-21126; M; IHC, FC, IF
Ubiquitin antibody (anti-mouse), Cell Signaling Technology, 3936; All; WB, IHC

The custom pS502 rabbit antibody was validated by comparing signal by immunoblot in WT and KO lines and by validating the loss of signal against a pospho-defective S502A mutant overexpressed TNK1 (see figure 1E). We also determined by immunoblot overlay that anti-mouse secondary antibody did not recognize the pS502 rabbit antibody (figure 1E, see overlay).

Eukaryotic cell lines

Policy information about cell lines

Cell line source(s): All cell lines were obtained from ATCC or DSMZ. Cell lines used: HEK-293T, Ba/F3, FDCP1, A549

Authentication: Cell lines were not authenticated after receipt from ATCC or DSMZ

Mycoplasma contamination: Cell lines have tested negative for mycoplasma

Commonly misidentified lines (See ICLAC register): No commonly misidentified cell lines were used in this study

Animals and other organisms

Policy information about studies involving animals; ARRIVE guidelines recommended for reporting animal research

Laboratory animals: Mus musculus, NOD-SCID, 6-8 weeks of age, males and females. Male and female mice were used in all experiments (male/female ratios distributed as evenly as possible between experiments). During the entire study period, animals were housed in IVC cages (individually ventilated cages). Cages were changed out to fresh sterile cages no less than every 2 weeks, or as often as necessary.

	Rooms used for this study were located at HCI. The rooms were illuminated by fluorescent lights on a 12 h light-dark cycle. Temperature and relative humidity in the rooms is controlled automatically and was monitored daily. The target ranges were 22°C ± 2°C for temperature and 15% - 40% for humidity. Mice had free access to food and water. The irradiated diet was supplied by ENVIGO (Cappecci Diet, 3980X). Main tap water (autoclaved before use) was provided ad libitum by watering bottles. For environmental enrichment, animals are provided with bedding packets for environmental enrichment to ensure adequate welfare and well-being.
Wild animals	This study does not involve wild animals
Field-collected samples	This study does not involve field-collected samples
Ethics oversight	IACUC, Huntsman Cancer Institute, University of Utah

Note that full information on the approval of the study protocol must also be provided in the manuscript.

Human research participants

Policy information about studies involving human research participants

Population characteristics	Patients of any age, race, or sex with a diagnosis or suspected diagnosis of a blood or bone marrow cancer are enrolled in this study. All specimens are de-identified after collection in order to blind the analysis.
Recruitment	Patients of any age with a diagnosis or suspected diagnosis of a blood or bone marrow cancer may be a part of this study. The provider who performs the blood draw, bone marrow aspirate, or biopsy as part of the patient's routine care identifies possible participants from their clinic schedule or patient list, gives them information about what would be involved in participating in the study including possible risks, and asks if they are interested in participating.
Ethics oversight	IRB, Oregon Health Sciences University

Note that full information on the approval of the study protocol must also be provided in the manuscript.

Clinical data

Policy information about clinical studies

All manuscripts should comply with the ICMJE guidelines for publication of clinical research and a completed CONSORT checklist must be included with all submissions.

Clinical trial registration	Clinical trial description available at https://clinicaltrials.gov, clinical trial number NCT01728402.
Study protocol	https://clinicaltrials.gov/ct2/show/NCT01728402?term=NCT01728402&draw=2&rank=1
Data collection	All data were collected at Oregon Health Sciences University. Trial enrollment is ongoing
Outcomes	This is a biorepository protocol. As such, the primary outcome measures are to Identify and determine the frequency of mutations causing aberrant signaling pathway function in patients with acute leukemias (AL), lymphoproliferative disorders (LPD), myelodysplastic syndromes (MDS), and myeloproliferative neoplasms. Integrated functional genomics studies (whole genome sequencing, *RNAi, proteomics, drug sensitivity, expression profiling) were also used to identify aberrant signaling pathways that contribute to the formation of hematologic malignancies.<br>*Relevant to this manuscript are the RNAi studies to determine tyrosine kinase dependency in patient cancer samples.
